# Cytocompatibility and Antibacterial Properties of Coaxial Electrospun Nanofibers Containing Ciprofloxacin and Indomethacin Drugs

**DOI:** 10.3390/polym14132565

**Published:** 2022-06-24

**Authors:** Shahla Khalili, Nazanin Ghane, Saied Nouri Khorasani, Fariba Heydari, Arjan Atwal, Pooya Davoodi

**Affiliations:** 1 Department of Chemical Engineering, Isfahan University of Technology, Isfahan 84156-83111, Iran; shahla.khalili65@gmail.com (S.K.); nazanin.ghane@ymail.com (N.G.); 2 Torabinejad Dental Sciences Research Center, School of Dentistry, Isfahan University of Medical Sciences, Isfahan 81746-73461, Iran; f_heydarie@dnt.mui.ac.ir; 3 School of Pharmacy and Bioengineering, Hornbeam Building, Keele University, Staffordshire ST5 5BG, UK; a.s.atwal@keele.ac.uk; 4 Guy Hilton Research Centre, Institute of Science and Technology in Medicine, Keele University, Staffordshire ST4 7QB, UK

**Keywords:** core–shell nanofibers, electrospinning, gelatin, cellulose acetate, poly (ε-caprolactone), antibacterial activity, drug release

## Abstract

A coaxial nanofibrous scaffold of poly (ε-caprolactone) and gelatin/cellulose acetate encapsulating anti-inflammatory and antibacterial drugs was co-electrospun for skin tissue regeneration. Indomethacin and ciprofloxacin as model drugs were added to the core and the shell solutions, respectively. The effect of the drugs’ presence and crosslinking on the scaffold properties was investigated. TEM images confirmed the core–shell structure of the scaffold. The fiber diameter and the pore size of the scaffold increased after crosslinking. The tensile properties of the scaffold improved after crosslinking. The crosslinked scaffold illustrated a higher rate of swelling, and a lower rate of degradation and drug release compared to the uncrosslinked one. Fitting the release data into the Peppas equation showed that Fickian diffusion was the dominant mechanism of drug release from the scaffolds. The results of biocompatibility evaluations showed no cytotoxicity and suitable adhesion and cell growth on the prepared core–shell structure. The antibacterial activity of the scaffolds was studied against one of the most common pathogens in skin wounds, where the existence of ciprofloxacin could prevent the growth of the *Staphylococcus* *aureus* bacteria around the scaffold. The obtained results suggested a new coaxial nanofibrous scaffold as a promising candidate for simultaneous tissue regeneration and controlled drug release.

## 1. Introduction

Regeneration of damaged skin tissue is still challenging due to the presence of pathogens at the wound site that jeopardize the restoration of skin layers, cause skin inflammatory responses, and consequently destroy the extracellular matrix at the wound site by their natural enzymes [1]. *Staphylococcus aureus* and *Pseudomonas aeruginosa* are the most common pathogens in skin wounds [2]. To reduce the risk of bacterial infection at the wound site and accelerate the wound healing process, an antimicrobial impregnated scaffold is needed. The continuous release of antimicrobial agents on the wound exterior provides long-term antimicrobial activity to rapidly eradicate pathogens [1]. To address this challenge, recent studies have mainly focused on designing skin tissue scaffolds exerting antibacterial and anti-inflammatory activities while providing substrates to favor cell growth and stratification [2,3,4,5,6]. In conformance with the literature, two drugs (i.e., ciprofloxacin and indomethacin) were selected for loading into and releasing from the electrospun nanofiber scaffold due to their antibacterial and anti-inflammatory activity, respectively.

Ciprofloxacin, a class of fluoroquinolone antibiotics with negligible side effects, has been chosen as a model drug for many activities, including the treatment of various infections such as skin and soft tissue infections, bronchitis, pneumonia, and urinary tract infections [1]. The mechanism of action of ciprofloxacin is such that after penetrating the bacterial cell membrane, it stops the synthesis of DNA in bacteria [6]. Indomethacin (C_19_H_16_ClNO_4_) is a non-corticosteroid anti-inflammatory drug generally used to moderate fever, pain, and swelling caused by inflammation. By blocking the effect of the enzyme, cyclooxygenase, indomethacin reduces the production of prostaglandin, which is responsible for causing pain and inflammation in the affected area [5].

Nanofibers containing various drugs have been used in the treatment of various types of cancer, diabetes, diseases related to the eyes, ears, brain, heart, and wound dressing [7,8,9]. Due to their porous structure, nanofibers can be a good substrate for cell growth and adhesion, and due to their high surface-to-volume ratio, they can increase cell adhesion, drug loading and release. Electrospinning allows for the direct encapsulation of any hydrophilic, hydrophobic, and biomacromolecular drug such as protein and DNA to fibers. Compared to other drug carriers such as hydrogels, liposomes, nanoparticles and micelles, electrospun nanofibers have the ability to improve drug storage efficiency and reduce abrupt release. Mixing the drug with a polymer solution is a common method of binding and storing the therapeutic agent in electrospun nanofibers. The resulting solution can be transformed into nanofibers by single-nozzle, multinozzle, or coaxial electrospinning [10,11]. Coaxial electrospinning, as an innovative modification of electrospinning, can be used for the encapsulation of drugs within polymer matrices with a core–shell nanofiber structure [12,13], where these electrospun nanofibers offer a similar structure to the native extracellular matrix (ECM). The native ECM is composed of protein-rich nanofibers in a polysaccharide matrix [14]. Gelatin is a collagen-derived protein which is widely used in tissue engineering applications [15,16]. Cellulose acetate (CA) is also a polysaccharide which has been widely used as wound dressing materials [17,18]. Literature has suggested Gelatin/CA (7/3) blend as a good candidate for simulation of the skin ECM [16,19,20].

This study aimed to prepare and characterize the core–shell nanofibers of gelatin/cellulose acetate and poly(ε-caprolactone) using the coaxial electrospinning method. Poly (ε-caprolactone) was used to reduce the drug release rate due to its low degradation rate [3,4,21]. Additionally, to improve the surface hydrophilicity of the scaffold, natural polymers of gelatin and cellulose acetate were used as the outer layer (shell) of the fibers to create proper signaling and interaction between the scaffold and the cell [19]. The indomethacin and ciprofloxacin as model drugs were added to the core and the shell solutions, respectively. The scaffold morphology and properties were studied. Additionally, the effects of the drugs’ presence and crosslinking of the shell components on the physical properties, drug release behavior, biocompatibility, and antibacterial activity of the scaffold were evaluated.

## 2. Materials and Methods

### 2.1. Materials

Poly(ε-caprolactone) (PCL, average M_n_ = 80,000 Da), cellulose acetate (CA, M_r_ = 29,000), and glutaraldehyde (GA, 25% *v/v*) as the crosslinking agent were obtained from Sigma Aldrich Company (Taufkirchen, Germany). Gelatin (Gel, type A from porcine skin) was obtained from Fluka, Switzerland. All solvents, including methanol, chloroform, and acetic acid aqueous solution, were supplied from Merck Company (Darmstadt, Germany). Indomethacin, as an anti-inflammatory drug, and ciprofloxacin, as an antibacterial agent, were purchased from DaruPakhsh Pharmaceutical Mfg. Co (Tehran, Iran). Culture media consisting of RPMI and fetal bovine serum (FBS) were bought from Gibco Company (MA, USA). Besides, 3-(4,5-dimethylthiazol-2-yl)-2,5-diphenyltetrazolium bromide (MTT), and penicillin-streptomycin were obtained from Sigma Aldrich Company, Taufkirchen, Germany.

### 2.2. Preparation of the Coaxial Nanofibers

As the core solution, a mixture of chloroform: methanol (4:1 (*v*/*v*)) was used to make a 7% (*w*/*v*) polymer solution of PCL. Then, indomethacin was added to the mixture at a concentration of 10% (*w*/*w*, drug/polymer). As the shell solution, a mixture of acetic acid: distilled water (9:1 (*v*/*v*)) was used to prepare a 20% (*w*/*v*) polymer solution of Gel/CA (7/3 (*w*/*w*)), according to previous studies [16,19,22]. Next, ciprofloxacin was added to this mixture at a concentration of 15% (*w*/*w*, drug/polymer). A coaxial electrospinning device (FNM Company, Tehran, Iran) consisting of two coaxial needles made of stainless steel was used. Co-electrospinning was performed at different flow rates, as shown in Table 1. A voltage of 13.5 kV and a distance of 13 cm were applied. Finally, the prepared nanofibrous scaffolds were placed in a vacuum oven for 24 h to remove residual solvents from the fibers. The morphology of the fibers was studied using scanning electron microscopy micrographs. Then, the selected scaffold was applied for the crosslinking process followed by characterization.

The optimized scaffold was crosslinked with GA vapor (25% v) at 40 °C for five hours and then washed with glycine solution (0.2 M) and phosphate-buffered saline (PBS) to remove any unreacted GA.

### 2.3. Characterizations of the Scaffold

#### 2.3.1. The Morphological Study

The electrospun core–shell fibrous samples and the crosslinked one were gold-coated for 10 min, and then applied for morphology characterization using scanning electron microscopy (SEM, Philips XL30, F.E.I. Company, Eindhoven, The Netherlands) at an accelerating voltage of 20 kV. Moreover, energy dispersive spectrum (EDS) mapping analyses were performed during SEM observation. The fiber diameters, pore size, and the porosity of the samples and the crosslinked one were determined by the SEM images using ImageJ (ImageJ, National Institutes of Health, Bethesda, MD, USA ) and using software (MATLAB, Mathworks, Inc., Natick, MA, USA). The mean values and the distributions were determined by IBM SPSS statistics software.

Transmission electron microscopy (TEM, Zeiss EM10C, 80 kV, Jena, Germany) was applied to confirm the core–shell structure of the optimized sample. The TEM sample was prepared by electrospinning on a carbon-coated copper grid.

#### 2.3.2. Fourier Transform Infrared Spectroscopy (FTIR)

Chemical characteristics of the optimized scaffold before and after crosslinking were determined using a Fourier transform infrared spectrometer (FTIR, Rayleigh WQF-510A, Beijing Beifen-Ruili Analytical Instrument Co., Beijing, China) using potassium bromide (KBr) pellets. A bare KBr pellet was considered as background, where its corresponding spectrum was subtracted from that of samples. The FTIR spectrums of the samples were recorded on transmittance mode in a range of 500–4000 cm^−1^ with a spectral resolution of 4 cm^−1^.

#### 2.3.3. The Tensile Property Evaluation

To compare the tensile properties of the uncrosslinked sample with the crosslinked one, a universal testing machine (Zwick 1446-60, Ulm, Germany) was applied to rectangular samples with equal dimensions (25 × 10 mm^2^). Before this test, the prepared scaffolds were placed in PBS for one hour, and their thickness was measured. Then, the tensile testing was performed with a 10 N load cell at a speed of 10 mm min^−1^ and a gauge length of 20 mm at room temperature.

#### 2.3.4. The Physical Properties Evaluation

The static water contact angle values of the scaffold before and after crosslinking were measured by a contact angle analyzer (CA-500A, Sharifsolar Co., Tehran, Iran). Three specimens were tested using 1 µL of water droplets. Then, the average contact angle values were calculated.

To compare the water uptake capability and the degradation ratio of the uncrosslinked sample with the crosslinked counterpart, pre-weighted samples (W_i_) (n = 3) with a diameter of 10 mm were submerged in PBS solution (pH = 7.4) at 37 °C. After 4 h and 1, 3, 5, 7, 14, 21, and 28 days, the scaffolds were removed from the PBS solution, and then weighted (W_s_), dried, and weighed again (W_d_). The PBS absorption percentage, and the weight loss (%) of the scaffolds were considered via the following equations [19]:

(1) $$\;\mathrm{PBS}\;\mathrm{absorption}\;\left(\%\right)=\frac{\left[\mathrm{Ws}-\mathrm{Wi}\right]}{\mathrm{Wi}}\times 100$$

(2) $$\;\mathrm{Weight}\;\mathrm{loss}\;\left(\%\right)=\frac{\left[\mathrm{Wi}-\mathrm{Wd}\right]}{\mathrm{Wi}}\times 100$$

### 2.4. Drug Release Study

In this study, the release of ciprofloxacin from the scaffolds was measured using a UV–VIS spectrophotometer (Beijing Rayleigh Analytical Instrument Corporation (BRAIC); Headquarters, Beijing, China) at the absorbance of 323 nm. The absorbance peak of the indomethacin was also at 318 nm with less intensity which overlaps with the ciprofloxacin peak. Before the drug release test, a standard calibration curve was plotted within the range of 13.64–30 μg mL^−1^ of ciprofloxacin in PBS. The calibration equation of the antibiotic drug was y = 28.86x − 2.0544, R^2^ = 0.9964. After that, at each time interval (explained in Section 2.3.4), 4 mL of each solution was taken out for analysis with the spectrophotometer, and their absorbance was recorded to obtain the concentration of the released drug from samples in the solutions (n = 3).

### 2.5. Biocompatibility Evaluation

Before cell seeding, the prepared samples (diameter of 15 mm) were placed in a 24-well plate and consequently sterilized with ethanol 70% (*v*/*v*), and UV light. The samples were then dipped in a culture medium (RPMI), supplemented with fetal bovine serum (FBS) (10% (*v*/*v*)), and penicillin-streptomycin (1% (*v*/*v*)), overnight.

All the cell culture protocols and the biocompatibility evaluations were performed according to the previous literature [19,20]. Briefly, the human normal gingival fibroblast cells (HGF1) were cultured in the RPMI culture medium, at 37 °C, under 5% CO_2_. The HGF was used as an example of fibroblast cells [19]. The cells with a density of 3 × 10^4^ cells per disc were seeded on the samples (n = 3). The culture medium was refreshed every three days.

The in vitro cell activity was studied by MTT assay. Briefly, the cultured cells on the uncrosslinked and crosslinked samples were incubated for 1, 3, and 7 days at 37 °C, 5% CO_2_, 90% relative humidity. At each time point, the cells were rinsed twice with PBS, and 40 µL MTT solution was added at 37 °C. After 3 h, 400 µL DMSO was added to each well to dissolve the purple crystals of formazan. The absorbance of each sample was measured at 570 nm using a spectrophotometer (Hiperion MPR4, Hyperion GmbH, Unna, Nordrhein-Westfalen, Germany). The tissue culture plate and DMSO were used as the background, and the UV absorbance of each group was calculated from the difference in the absorbance of the group with the DMSO one [23,24,25].

Morphology of the HGF cells were observed using SEM. After 1 day and 7 days of cell seeding, the uncrosslinked and the crosslinked samples were washed with PBS, fixed with 2.5% (*v*/*v*) glutaraldehyde for 1 h, and dehydrated in different concentrations of ethanol (70, 80, 90, and 99.8% *v*/*v*). The scaffolds were finally dried and gold coated before SEM analyses.

### 2.6. Antibacterial Activity

Antibacterial activity of the samples against the Gram-positive bacterium *Staphylococcus aureus* (ATCC 25923) were assessed by the disk diffusion method. The 0.5 McFarland bacteria were cultured on plates containing a blood agar medium. The scaffold discs before and after crosslinking, along with paper discs containing 75 and 150 μg of ciprofloxacin, and blank discs (sterile discs impregnated with distilled water), were placed on the plate. Afterward, plates were incubated at 37 °C for 24 h. Then, the inhibition zone for each sample was evaluated [26,27].

### 2.7. Statistical Analysis

All experiments were performed in triplicate, and the obtained results were reported as mean ± standard deviation. The various groups were compared using a one-way analysis of variance (ANOVA) using SAS 9.4 (Virginia SAS Users Group (VASUG), Richmond, VA, USA) and a *p*-value < 0.05 was considered to be statistically significant.

## 3. Results and Discussion

### 3.1. Optimization of Co-Electrospinning Conditions

**Figure 1:**
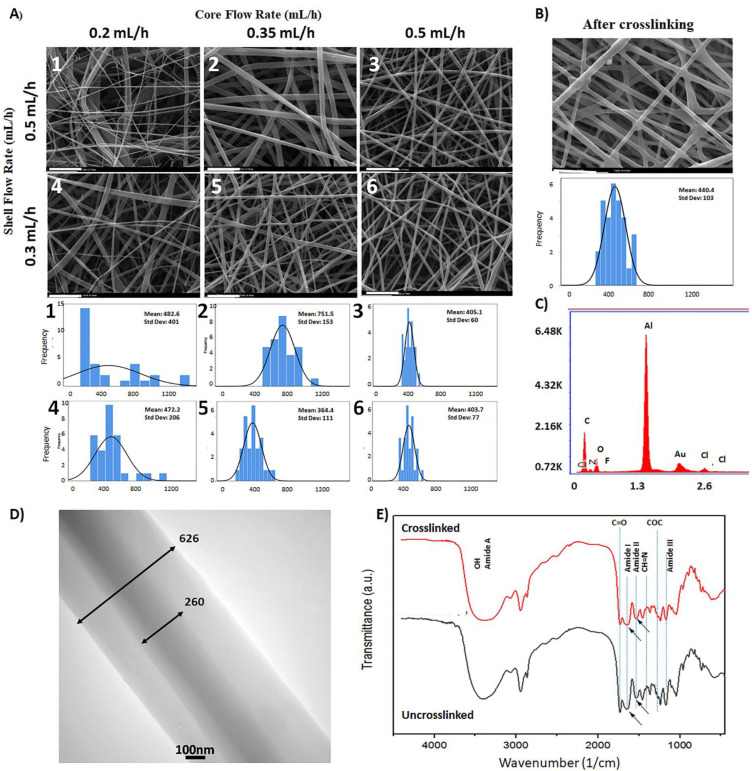
The morphological and chemical study; (**A**) the SEM micrographs (scale bar = 5 μm), and the fiber diameter distribution of the prepared samples at different flow rates of the core and shell solutions; (**B**) the SEM micrograph (scale bar = 5 μm) and fiber diameter distribution of the optimized scaffold after crosslinking; (**C**) the EDX spectrum of the prepared scaffold; (**D**) the TEM image of the prepared core–shell nanofiber; (**E**) the FTIR of the prepared scaffolds before and after crosslinking.

One of the influential parameters in the electrospinning process is the flow rate of the solution, which determines the transfer rate of the solution from the nozzle to the collector. The time required for solvent evaporation is provided by selecting the appropriate flow rate [28]. The coaxial electrospinning of the solutions was performed by changing the flow rate of the shell solution from 0.3 to 0.5 mL h^−1^, in different values of the core flow rate, including 0.2, 0.35, and 0.5 mL h^−1^, according to Table 1. The SEM micrographs of the samples and the fiber diameter distribution are shown in Figure 1A. As shown, by increasing the flow rate of the core solution from 0.2 to 0.5 mL h^−1^, more uniform fibers with narrower fiber diameter distribution were attained. This trend was observed for both shell flow rate values. In other words, by increasing the core flow rate, the fibers were transformed from a strip-shaped and non-uniform state to fibers with a circular cross-section and a uniform size distribution. The higher contribution and consequently the higher strength of PCL compared to the shell constituents could be the reason for this observation [21].

The average diameter of the fibers and pores of the scaffolds and the surface porosity are presented in Table 1. The diameter of the fibers ranged from 364 to 752 nm, and the pore size of the scaffolds varied from 2.3 to 4.5 μm. In general, fibers with larger diameters create larger pores in the scaffold [19,20,29]. The surface porosity of the prepared scaffolds was determined using SEM images. As shown in Table 1, all prepared scaffolds showed a surface porosity of more than 87%.

The narrowest diameter distribution, the best fiber quality, and the lowest fiber diameter were observed in samples 3 and 6 with a core flow rate of 0.5 mL h^−1^. Between these two samples, sample 3 with the flow rate of 0.5 mL h^−1^ for the core and shell was selected for further study. The higher flow rate of the shell increases the chance of loading more antibiotics into the shell structure. Therefore, the prepared scaffold was electrospun under optimal conditions and then crosslinked by glutaraldehyde vapor for five hours. Afterward, the scaffold was washed with glycine solution and PBS to remove any unreacted materials, and finally, it was dried.

### 3.2. Morphology of the Scaffolds after Crosslinking

**Scheme 1:**
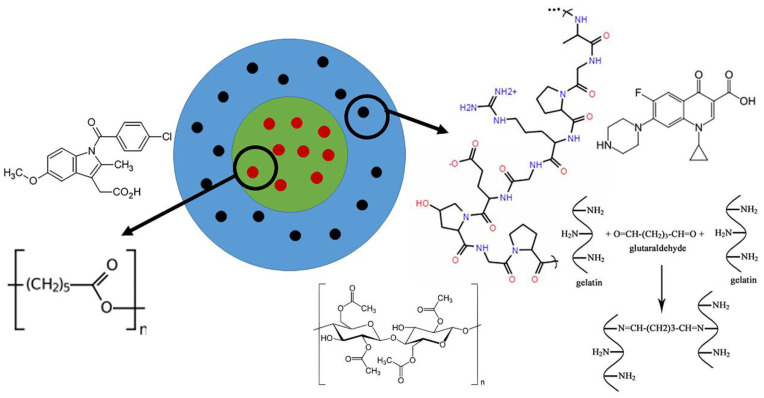
A schematic of the core–shell structure with the respective components involved in the formation the scaffold.

In this study, a glutaraldehyde solution (25% v) was used as a crosslinking agent. Glutaraldehyde is a clear, oily 5-carbon dialdehyde and has received much attention due to its high reactivity and reasonable price [30,31]. The reaction between the amine groups of gelatin and the aldehyde group in glutaraldehyde leads to the reduction in hydrophilic groups and thereby reduces the hydrophilicity of the scaffold (Scheme 1) [32].

Figure 1B shows the SEM micrograph and the fiber diameter distribution of the optimal sample after crosslinking. The crosslinking made significant changes in the morphology, fiber diameter, pore size, and porosity of the scaffold. The fused fibers with many intersection areas were attained after crosslinking. The mean fiber diameter increased from 405 ± 60 to 440 ± 103 nm, which was not statistically significant (*p* > 0.05). The pore size of the scaffold was increased from 2.6 to 5.5 μm during the crosslinking process, due to the fusion of the fibers together. Other researchers also reported an increase in the fiber diameter and pore size of the scaffolds during crosslinking with glutaraldehyde vapor [19,33,34,35]. Increasing the pore size of the electrospun scaffolds for tissue engineering applications is an important issue that has received much consideration in the last decade. Larger pores allow for the efficient transport of nutrients and waste within the scaffold and facilitate the migration of cells inside the scaffold [20,36,37]. The surface porosity of the scaffold also decreased from 88% to 84% during the crosslinking process (*p* > 0.05). Grover et al. also reported a reduction in porosity from 90% to 80% during the crosslinking of the gelatin/elastin films with EDC and NHS [32].

The energy dispersive X-ray (EDX) was also performed to investigate the presence of ciprofloxacin and indomethacin inside the scaffold structure. As shown in Figure 1C, the presence of chlorine and fluorine peaks confirms the existence of these two drugs in the structure. Due to the structure of ciprofloxacin (C_17_H_18_FN_3_O_3_), it was expected to observe fluorine atoms in addition to carbon, oxygen, and nitrogen atoms, which are also present in the structure of the constituent polymers [2]. Similarly, the presence of chlorine atoms confirmed the presence of indomethacin (C_19_H_16_ClNO_4_) in the fiber structure [38]. It should be noted that the gold and aluminum peaks are related to the gold coating on the surface of the sample and the aluminum foil under the sample, on which electrospinning was performed.

### 3.3. The Core–Shell Structure of the Prepared Scaffold

To confirm the core–shell structure of the prepared fibers, transmission electron microscopy was used. For this purpose, electrospinning was performed directly on the copper mesh plate in the optimal conditions. As demonstrated in Figure 1D, coaxial electrospinning of gelatin/CA and PCL solutions produced a core–shell structure where the core diameter was 260 nm and the outer shell diameter was 626 nm.

### 3.4. The Chemical Structure of the Core–Shell Scaffold

The FTIR spectrum was used to investigate the chemical structure of gelatin, CA, and PCL, and to confirm the crosslinking of the gelatin shell layer (Figure 1E and Scheme 1). The pure gelatin spectrum includes specific peaks at 1645, 1540, 1240, and 3300 cm^−1^ for amide I, II, III, and amide A, respectively [21,39,40]. Characteristic peaks of cellulose acetate also include 1745 cm^−1^ (C=O), 1240, 1160, and 1049 cm^−1^ of C-O-C group, and the broad peak of -OH group between 3000–3700 cm^−1^ [17,19,41].

Characteristic peaks of PCL include two asymmetric and symmetric peaks of the methylene group (2944 and 2867 cm^−1^), a strong peak at 1720 cm^−1^ (C=O), and 1471 and 1419 cm^−1^ (methyl group), and 1397 and 1365 cm^−1^ of CO [42,43].

The FTIR spectrum confirmed the presence of all three polymers in the scaffold. The characteristic peaks of both drugs were in the same range as the polymers and could not be detected due to overlap. However, as observed, the EDX spectrum confirmed the presence of both drugs in the structure.

In addition to the mentioned peaks, a strong peak observed in 1400 cm^−1^ is related to the aldimine group in the crosslinked scaffold. The color change in the scaffolding during the crosslinking process from white to yellow also confirms the formation of an aldimine group (CH=N) within the structure. Additionally, the decrease in the intensity of amide I and II bands, related to the gelatin structure in the crosslinked sample compared to the uncrosslinked one, indicates the reduction in the number of amine groups due to the crosslinking [19,44].

### 3.5. The Tensile Properties of the Core–Shell Scaffold

**Figure 2:**
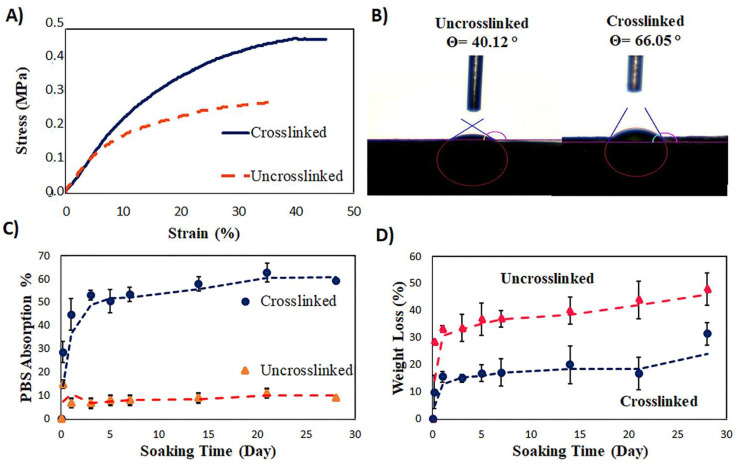
The mechanical and physical characterizations; (**A**) the tensile stress–strain curve; (**B**) the images of the water contact angle test; (**C**) the PBS absorption (%); (**D**) the weight loss (%) of the coaxial scaffolds before and after crosslinking.

The tensile test was performed in the wet condition to better simulate in vivo conditions, and the tensile modulus, stress, and elongation at the break of the specimens were calculated. The stress–strain curve of the samples is shown in Figure 2A, and the obtained results are reported in Table 2. After crosslinking, the tensile modulus of the scaffold increased to some extent (*p*-value = 0.06), and tensile strength and elongation at break also showed a significant increase (81% and 22%, respectively). The improved tensile property of the scaffold is due to the creation of strong networks between and within the fibers. A similar trend has been observed in other studies. Grover reported Young’s modulus and elongation at break of the pure gelatin at 4.6 kPa and 27% [32]. Vatankhah et al. reported the tensile strength and elongation at break of the gelatin/cellulose acetate nanofibers of 1.59 MPa and 5.14%, respectively, which is very close to the skin properties [16]. In the previous study, Young’s modulus, tensile strength, and strain at break of the gelatin/cellulose acetate sample were reported to be about 0.6 MPa, 0.37 MPa, and 40%, respectively [19,20]. It was expected that the presence of PCL in the core structure, with higher modulus and strength than gelatin and cellulose acetate, increases the tensile properties of the core–shell scaffold. A similar tendency was reported in the literature [3,21].

### 3.6. The Surface Hydrophilicity of the Coaxial Scaffold

The hydrophilicity of the scaffold is an essential parameter in cell adhesion to the scaffold. To investigate the crosslinking effect on the hydrophilicity of the scaffold surface, a static contact angle test was performed on the scaffold before and after crosslinking. As shown in Figure 2B, the contact angle of the uncrosslinked scaffold is about 40°, which is significantly lower than that of the crosslinked scaffold (66°). The presence of hydrophilic groups, i.e., -COOH and -NH_2_, in the gelatin structure reduces the contact angle of the scaffold and increases the wettability of the scaffold surface. However, the consumption of the amine groups during the crosslinking process increases the contact angle of the crosslinked scaffold [30,31].

### 3.7. The Swelling Ratio and Biodegradation of the Coaxial Scaffolds before and after Crosslinking

The swelling of the scaffold in the physiological environment is an important feature in tissue engineering as it is related to the bulk hydrophilicity of the scaffold. The swelling facilitates nutrient transport and cell infiltration within the scaffolds and improves the absorption of wound secretions. While excessive swelling of the scaffold exerts undesirable pressure on the surrounding tissues, insufficient swelling undermines cell adhesion and proliferation [19]. The swelling behavior of the scaffolds was studied after immersion in PBS solution over 28 days. Figure 2C shows the swelling percentages of the scaffold following PBS absorption. The swelling of the crosslinked scaffold increased by 53% by the third day and then reached equilibrium. The maximum swelling was about 60% after 28 days in this scaffold. The PBS absorption of the uncrosslinked scaffold also initially showed a 15% increase in the first 4 h. However, the PBS uptake in the sample decreased to 8% and then showed a balanced behavior over time. As observed, the uncrosslinked scaffold had higher surface hydrophilicity than the crosslinked one. Additionally, the fiber diameter of the uncrosslinked scaffold was smaller than the crosslinked scaffold. Therefore, it is expected that the infiltration of the solution into the uncrosslinked fibers occurs faster and to a greater extent. However, the weight gain of the uncrosslinked sample is considerably less than that of the crosslinked sample, which is due to the gelatin degradation and dissolution of the uncrosslinked scaffold in PBS solution [45,46]. As seen in Figure 2D, both scaffolds showed severe weight loss until the first day and then until the 28th day of immersion in PBS solution with a much lower slope. The degradation rate in the uncrosslinked sample was almost twice that of the crosslinked scaffold. Degradation of the scaffolds is accomplished by hydrolysis of amino acid groups in gelatin, and decomposition of the ester bonds in cellulose acetate. Before crosslinking, the amount of amino acid groups in the structure is higher. On the other hand, the diameter of the fibers in the uncrosslinked scaffold were smaller than the other one. Additionally, the porosity of the scaffold was reduced during the crosslinking process. Therefore, the required time for the PBS solution to penetrate into and between the fibers increased and the degradation rate decreased due to crosslinking. Grover et al. reported the degradation of 25% in crosslinked gelatin/elastin scaffolds after 14 days [32]. In this study, the weight loss percentage of the crosslinked sample changed from 15% on the first day to about 31% after 28 days, whereas in the sample before crosslinking, the degradation percentage was 33% on the first day and increased to about 48% after 28 days. Therefore, the shell layer was almost degraded after 4 weeks, which can result in rapid and uncontrolled release of ciprofloxacin at this time period.

### 3.8. The Drug Release Study

**Figure 3:**
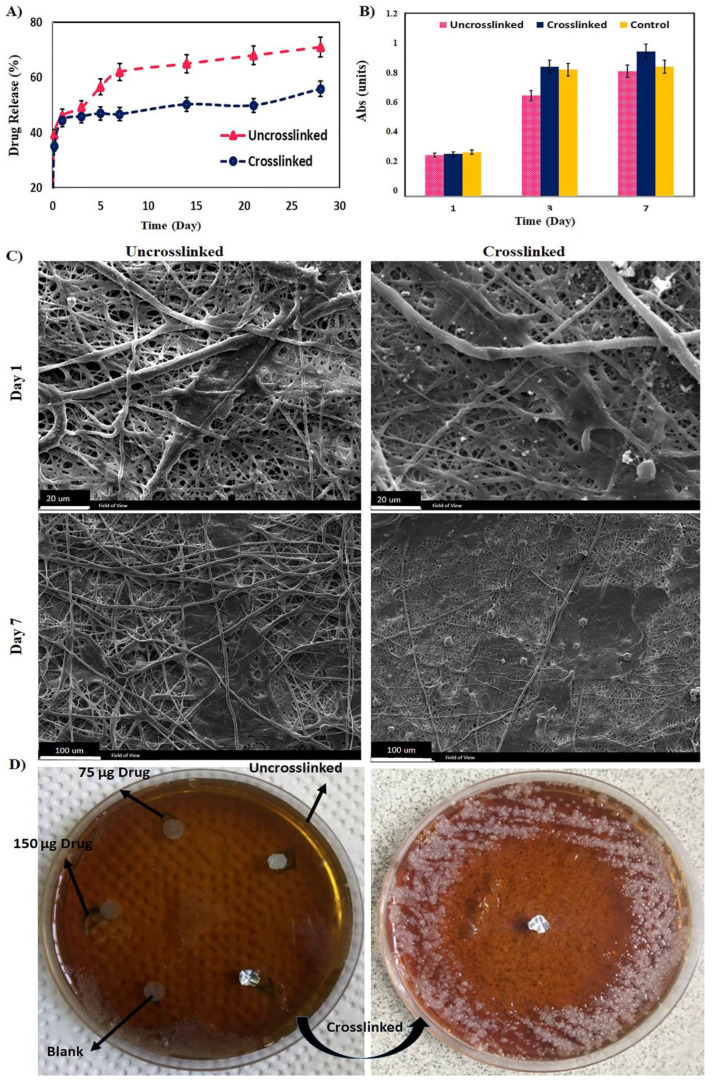
(**A**) The cumulative release of ciprofloxacin from the scaffolds before and after crosslinking; (**B**) the MTT results; (**C**) the SEM micrographs of the scaffolds (scale bar = 20 and 100 μm); (**D**) the antibacterial activity of the prepared scaffolds.

The initial loading of the ciprofloxacin in the shell solution was 15% (*w*/*w*, drug/polymer). The drug concentration released from the scaffold before and after crosslinking was measured using a UV-Vis spectrophotometer in the range of 200–800 nm. Maximum absorption of ciprofloxacin was measured at 323 nm. The standard calibration curve was drawn in the range of 13.64 to 30 μg ciprofloxacin/mL PBS. The calibration equation was calculated as y = 28.86x − 2.05, R^2^ = 0.9964. The linearity of the standard calibration curve and its correlation coefficient confirms the Beyer–Lambert law. Figure 3A shows the cumulative drug release (%) from the scaffold before and after crosslinking over 28 days. Both samples showed an initial rapid release and a lower release rate similar to the degradation results. Therefore, the rapid initial release can be attributed to the initial dissolution and degradation of gelatin in the shell. The rate of drug release in the uncrosslinked sample is slightly higher than that in the crosslinked one, confirming the faster degradation of the uncrosslinked sample. According to the results, about 50, and 70% of the loaded ciprofloxacin in the shell structure was released over 28 days from the crosslinked, and the uncrosslinked scaffold, respectively. Therefore, crosslinking was effective in controlling the drug release, and decreasing the release rate of the drug from the scaffold [15].

- Kinetics and Mechanism of the Drug Release

To determine the kinetics and mechanism of the drug release, the proposed Peppas Equation was used. The obtained results from the release of ciprofloxacin fitted the equation.

(3) $$\;\;\;\;\;\;\;\;\;\;\;\;\;\;\;\;\;\;\;\;\;\;{\;\mathrm{kt}}^{n}=M_{t}/M_{e\;}$$

In this equation, M_t_ is the amount of drug released at any given time, and M_e_ is the equilibrium amount of the released drug. The value of n (drug diffusion coefficient) determines the mechanism of the drug release; the value of 0.5 correlates to the Fickian diffusion, and the value of one represents non-Fickian diffusion (swelling influence) [4,47].

The drug release mechanism from the scaffolds was also adapted to the other proposed Peppas models, including the zero-order, first-order, and Higuchi models. The correlation coefficient (R^2^) of different models (Table 3) was used to investigate the best fitting model to describe the mechanism of drug release from the scaffolds.

According to the results obtained from curve fitting, the n value for both scaffolds was about 0.074, which shows a Fickian diffusion behavior of the drugs released from the scaffold. Additionally, the highest correlation coefficient was related to the Higuchi model. When the drug release rate from the polymer matrix is higher than the rate of polymer degradation and diffusion occurs through the pores of the structure, the Higuchi model is more consistent [4,43,48]. Therefore, due to the high hydrophilicity of the ciprofloxacin and the shell polymers, the release of the drug from the scaffold follows the Higuchi model [4,38].

### 3.9. The Biological Study

To estimate the biological activity of the prepared scaffolds, human gingival fibroblast cells (HGF1) were cultured on the scaffolds and examined for seven days. Cell metabolic activity was assessed using an MTT assay. The living cells on the scaffolds convert the MTT compound into a pigment called formazan. The absorption of formazan at 570 nm is directly correlated to the number of living cells in the scaffold. Figure 3B demonstrates the absorption value at this wavelength for the scaffolds compared to the control sample (cell culture plate). As shown, the amount of absorption in both samples was not significantly different from the control sample (*p*-value = 0.09). In all samples, the absorption value increased from day one to day seven. These results confirmed that the samples had no cytotoxicity and cells grew well on the surface of the scaffolds. The amount of absorption in the crosslinked sample is not significantly different from the other samples, meaning that crosslinking did not cause toxicity.

Cell morphology was also evaluated using SEM images. Figure 3C shows the micrographs of the scaffolds taken before and after crosslinking, 1 and 7 days after cell seeding. Cell adhesion and morphology on both scaffolds were normal where hydrophilicity, as well as the appropriate chemical structure of the shell polymers, has increased the tendency of the cells to adhere to these scaffolds. On the other hand, larger pores in the crosslinked scaffold helped cells to migrate into the fibrous structure. Previous studies also showed that cells are more likely to penetrate structures with larger pores [29].

### 3.10. The Antibacterial Study

The antibacterial activity of the samples was evaluated by measuring the inhibition zone of *Staphylococcus aureus* in the disc area (Figure 3D). The average diameter of the inhibition zone for each sample is presented in Table 4. As shown, the diameter in both scaffolds was close to the zone diameter of the control sample containing 75 micrograms of ciprofloxacin. Therefore, it can be concluded that the actual loaded amount of the drug is close to this amount, and the optimum concentration would be lower than 75 µg. Overall, both samples showed suitable antibacterial activities against the Gram-positive *Staphylococcus aureus* [27]. The bigger inhibition zone around the crosslinked samples compared to the one with uncrosslinked scaffolds can be due to the washing of the ciprofloxacin during the preparation and sterilization stage. It should be noted that indomethacin has milder antibacterial activity compared to ciprofloxacin. Sukul et al. reported the inhibitory zone of *Staphylococcus aureus* for indomethacin and ciprofloxacin drugs as 9 and 45 mm, respectively [49]. Therefore, the antibacterial activity of the prepared scaffolds is more related to the presence of ciprofloxacin in the structure.

The results showed no cytotoxicity and no bacterial growth in both samples, so the presence of the drugs in the structure of the scaffolds had no adverse effect on the growth of fibroblast cells and well prevented the growth of *Staphylococcus aureus* bacteria around the scaffold. As mentioned, *Staphylococcus aureus* is one of the most common pathogens in skin wounds that can destroy the extracellular matrix at the wound site and delay tissue regeneration. The data showed that the scaffolds encapsulating highly active, antibacterial drugs can efficiently prevent the growth of bacteria. Furthermore, crosslinking improved the tensile properties of the scaffold close to the skin tissue properties, due to the creation of strong networks between and within the fibers. Additionally, crosslinking increased the PBS absorption which translates to better absorption of wound secretions. Besides, crosslinking of the prepared scaffold prevented the fast degradation of the fiber shell and provided a slower drug release [26,27]. Overall, a new coaxial nanofibrous scaffold containing antibacterial and anti-inflammatory drugs was prepared with similar properties to skin tissue. The scaffold provides appropriate antibacterial activity, acceptable cell attachment and proliferation, and controlled drug release. Therefore, it can be considered a promising candidate for simultaneous tissue regeneration and controlled drug release.

## 4. Conclusions

In this study, a core–shell scaffold of gelatin/cellulose acetate-poly (ε-caprolactone) containing two drugs, ciprofloxacin and indomethacin, was prepared. First, the flow rate of the core and shell solutions were investigated and optimized to achieve nanofibers with a smaller diameter and the best morphology. Increasing the flow rate of the core solution from 0.2 to 0.5 mL·h^−1^ improved the quality and uniformity of the fibers. This trend was observed for both shell flow rates (0.3 and 0.5 mL·h^−1^). The diameter of the fibers ranged from 364 to 752 nm, and the pore size of the scaffolds ranged from 2.3 to 4.5 μm. According to the obtained average diameter, diameter distribution, and fiber morphology, the same flow rate of 0.5 mL·h^−1^ was selected for further studies. The EDX analysis confirmed the existence of both ciprofloxacin and indomethacin in the structure. The TEM images confirmed the production of scaffolds with a core–shell structure. FTIR results showed the presence of polymer components in the scaffold structure and successful crosslinking of fibers. The resulting fibers were crosslinked using glutaraldehyde vapor to control the swelling behavior and degradation rate. The tensile modulus of the scaffold increased somewhat after crosslinking, while the tensile strength and strain at break increased significantly. The crosslinked scaffold showed higher bulk hydrophilicity, and lower degradation and drug release rates compared to the uncrosslinked scaffolds. The results obtained from matching the drug release data with the Peppas equation showed Fickian diffusion as the dominant mechanism of drug release from the scaffold. In vitro cell culture experiments confirmed the non-toxic effect of the scaffolds before and after crosslinking. Finally, the encapsulation and controlled release of ciprofloxacin from the structure prevented the growth of bacteria that damaged the extracellular matrix at the wound site. Therefore, the prepared core–shell structure with similar properties to the skin ECM provided appropriate antibacterial activity, good biological response and controlled drug release.

## Figures and Tables

**Table 1:** The electrospinning condition, fiber diameter, pore diameter, and surface porosity of prepared scaffolds.

Sample Code	Shell Flow Rate (mL·h^−1^)	Core Flow Rate (mL·h^−1^)	Fiber Diameter (nm)	Pore Diameter (µm)	Surface Porosity (%)
1	0.5	0.2	482 ± 401	3.0 ± 0.1	87
2	0.5	0.35	752 ± 153	4.5 ± 0.2	87
3	0.5	0.5	405 ± 60	2.6 ± 0.1	88
4	0.3	0.2	472 ± 206	2.3 ± 0.1	91
5	0.3	0.35	364 ± 111	2.9 ± 0.1	89
6	0.3	0.5	404 ± 77	2.8 ± 0.1	87

**Table 2:** The tensile modulus, strength, and elongation at break of the core–shell scaffold before and after crosslinking.

	Property	Tensile Modulus (MPa)	Tensile Strength (MPa)	Elongation at Break (%)
Sample				
**Before crosslinking**		1.85 ± 0.2	0.26 ± 0.02	36 ± 4
**After crosslinking**		2.4 ± 0.3	0.47 ± 0.04	44 ± 1

**Table 3:** The correlation coefficient (R^2^) was obtained from different models fitted to the drug release data.

Scaffold	Peppas Eq.	Zero-Order Eq. C_t_ = C_0_ + K_0_t	First-Order Eq. dC/dt = −K_1_t	Higuchi Eq. Q = K_H_ t ^1/2^
**Uncrosslinked**	y = 0.6117x^0.0746^ R^2^ = 0.9708	y = 0.0095x + 26.989 R^2^ = 0.8222	y = −0.0001x + 1.8917 R^2^ = 0.3084	y = 0.2905x + 25.385 R^2^ = 0.9272
**Crosslinked**	y = 0.6x^0.073^ R^2^ = 0.8673	y = 0.0202x + 34.461 R^2^ = 0.4569	y = −0.0002x + 1.8717 R^2^ = 0.4582	y = 0.7114x + 29.845 R^2^ = 0.6739

**Table 4:** The average diameter (mm) of the inhibition zone around the prepared scaffolds.

Control Disc	Disc with 75 µg Drug	Disc with 150 µg Drug	Uncrosslinked Scaffold	Crosslinked Scaffold
0	29.5 ± 0.03	30.3 ± 0.02	27.4 ± 0.01	27.9 ± 0.01

## Data Availability

Not applicable.
